# A Course of Clinical Lectures, Delivered at the Hôtel Dieu, Paris, for the Session 1842-’43

**Published:** 1843-10-28

**Authors:** A. F. Chomel

**Affiliations:** Paris


#THE MEDICAL EXAMINER,

### And Retrospect of the Medical Sciences.

Vol. VI.] PHILADELPHIA, SATURDAY, OCTOBER 28, 1843. [No. 21.

### COURSE OF CLINICAL LECTURES,

*Delivered at the Hôtel Dieu, Paris, for the Session* 1842–'43.

BY A. F. CHOMEL, M. D.

### LECTURE XIV.

RESUMÉ OF THE ANNUAL CLINIC.

(Continued.)

### RHEUMATISM.

During the last year thirty-two cases of rheumatism have entered the wards, of which twenty-three occurred in the summer semester, and nine only in the winter. The contingent furnished by the women was nearly double that of the men; twenty for the former, and eleven for the latter. A large number of the patients were over forty years of age; there were very few under twenty. The majority of cases were relapses; three only were primitive. In a few one joint only was affected, but in the greater number the affection was ambulant, passing successively from one articulation to another; in many all the joints were affected at the same time. I have sought for the probable causes of the disease in all these various patients. The opinion generally received among physicians, ancient as well as modern, is that cold plays a conspicuous part in the production of this disease. Giacomini, a distinguished Italian physician, has greatly insisted on this cause. Facts, however, do not always confirm this opinion; or, rather, we are not always able to discover the relation which exists between the cause and the effect. In eight of our patients humidity and cold were recognised as influencing the production of the disease, or, at least, as a predisposing circumstance. In eight others there was very positive exposure to cold, preceding a short time the morbid phenomena, and which doubtless contributed notably to them. In the other cases sometimes humidity, sometimes suppression of the catamenia, and in two cases the post-puerperal condition seemed to develope the attack, more or less immediately. Finally, thirteen times it was impossible to determine an appreciable occasional cause; this is more than one-third of the number of cases of rheumatism this year—a very large proportion. This proves that this affection is, like many others, under the influence of an intense predisposition, of a constitutional cause, and that its progress and developement are often independent of any exterior cause; so that you should not, consequently, consider it as an ordinary plegmasia, but as *specific inflammation sui generis.*

Rheumatism is usually ushered in with a chill; this occurred in about one-half the cases. Those who did not mention this symptom, did not probably recollect what they experienced at the commencement.

In twenty-four cases the disease attacked the joints of both the inferior extremities simultaneously. Once only did a single articulation suffer.

I am now going to consider a very important question, that, namely, of the complication of rheumatism with a morbid state of the heart, a complication which has been so much discussed within a few years past. The phenomena presented by auscultation in this disease are of great interest, inasmuch as they serve to establish what, probably, without this means of investigation, would have remained unperceived. But the manner of interpreting these phenomena, and explaining their relations with organic lesions of the heart, may lead to great errors. Thus, for example, when an anormal sound exists in the heart of a patient subject to rheumatism, the existence of a lesion of the organ need not necessarily follow. Autopsies of subjects who have died of rheumatism, or other affections complicating rheumatism, and in whom during life the existence of abnormal cardiac sounds have been ascertained, showed that in such cases there was very often no trace of disease of the heart. We may infer then that the semeiotic value of these morbid sounds is not yet perfectly appreciated; the perception of these sounds is, doubtless, useful as a sign of an abnormal phenomenon, but you should not lose sight of the fact also that these sounds may exist in the organ independent of any organic lesion. This being understood, we now arrive at the question of the coincidence of certain anormal sounds in the heart, and rheumatism.

Out of thirty-two rheumatismal patients, there were fourteen in whom there was a bruit de souffle, more or less marked, accompanying the first sound. In three, one of whom died, the pericardiac [friction] sound was heard. In fifteen cases, that is to say in nearly one-half, there was no abnormal bruit; two out of these fifteen died; the rheumatismal affection was not very marked in these two cases. In one of the two, however, a young woman twenty two years old, the symptoms were rather more positive. At her entrance she offered all the symptoms of chronic bronchial catarrh; she had crackling at the base of the lung, cough with dyspnœa, &c., she had at the same time pains in the articulations. She was bled six times, and large doses of tartar emetic were administered. In spite (?) of this active treatment, she succumbed. At the autopsy we found in the synovial membrane of the affected joint, a quantity of synovia greater than in the normal state, with some albuminous floculi; there was neither redness, nor any other sign of genuine phlegmonous inflammation. The lung presented all the physical signs of hepatisation, the bronchiæ being likewise inflamed. In the pericardium there was some serosity, a little cloudy, and some traces of injection, but no appearance of genuine phlegmasia. The chief seat of disease then, in this woman, was the lungs, which was in perfect accordance with the symptoms noticed during life.

The second case, which terminated in death, was that of a man in whom pus was found in one of the joints. Was this pus the product of a rheumatismal affection? This man previous to his entrance at the Hôtel Dieu, remained sometime at the Charité, to be treated for several abscesses in the feet; the purulent collections were opened, and the patient seemed to be cured; pain was subsequently felt in the knee of the same side, which being attributed to rheumatism, he was sent to this service. On his arrival the patient gave us a very imperfect history of what he had previously suffered, but declared to us that he never had had rheumatism. You should remark that some time before this affection he had felt pain in the other knee. In the course of the disease he was attacked with severe pneumonia and died. At the autopsy we found synovia in the right knee in larger quantity than usual, but pure synovia and nearly natural. The left knee contained purulent serosity. The existence of this pus in the knee should not furnish a very conclusive argument in favor of the possibility of the termination of rheumatism in suppuration. In fact when you find purulent deposits pre-existent to a rheumatismal affection, can we say that these deposits are due to rheumatismal inflammation? Long before the work of Dr. Latour you will find numerous cases of muscular rheumatism terminating in suppuration, reported. This termination of rheumatism was then generally believed in. In my inaugural dissertation I discussed at length these cases, and will not recur to them here. I will content myself with remarking that often phlegmons are developed in the neighborhood of articulations, and which are accompanied with pain, heat, fever, and all other symptoms belonging to the phlegmasiæ. Now, it is cases of this kind that are taken for articular rheumatisms, and these cases terminating in suppuration, this termination is referred to rheumatism, and not to the phlegmasia. This occurs oftener than we suppose; they have been the object of new researches, and they have been grouped under the head of articular rheumatism; but, all these facts I consider as exaggerations or as errors of diagnosis. Thus, a great number of these pretended cases of purulent rheumatism are sometimes phlebitis, with metastatic abscess of different organs and sometimes a genuine purulent diathesis. What is there then astonishing that pus has been found in the articulations?

In women recently delivered you see numerous abscesses developed in the articulations, as well as in other parts of the body, without the patient's having experienced any pains scarcely. These cases belong to the same group as the preceding; a purulent diathesis is always involved. At the same time that pus is found in the joints, you find purulent collections in other parts of the body, in the subcutaneous cellular tissue, for example; these abscesses cannot evidently be attributed to a rheumatismal cause, and why should the pus found in the joints have this origin? Why admit two different causes for effects of the same nature? There still exist two other causes of suppuration in articular rheumatism. One is the immoderate exercise which many persons suffering from rheumatism indulge in, exercise very often required from the nature of their occupation. In such cases, there is no doubt but that pus may form in the affected joints, but this suppuration is nothing more than an irritation consecutive to the constant motion of the limb; it is the result of an acute arthritis succeeding simple rheumatism. You conceive, consequently, that these cases prove little. In many of them upon which they argued, synovia alone and not pus was found in the articular capsule. Finally, many of them are so meager in sufficient details, that no inferences can be drawn from them.

*Treatment.*—I have not spoken of chronic rheumatism, but only of acute rheumatism with febrile re-action. I have treated this affection as an inflammation *sui generis*. In the treatment, we have used at the commencement, general and local bleeding, when the individual circumstances permitted it. These bleedings were followed by emollient fomentations, diaphoretic drinks and sometimes purgatives, according to the indications. Our attention was always especially directed to the complications, for these complications often occur in the lungs and brain; in such cases it is the pneumonia and the meningitis which should occupy us, as the graver affection. I do not share the opinion of those physicians who employ in such cases cold drinks; I prefer giving them blood warm. The diet should be very strict. Neither do I employ those heroic bleedings, *coup sur coup* in a single day; for the results obtained by this method, do not differ essentially from those obtained by the ordinary phlogistic treatment. In comparing patients treated by the two methods, I have found the same mean—18 days—for the duration of the disease. It is not necessary, therefore, to resort to these copious venesections, when moderate ones will suffice. The diaphoretic treatment I do not think a suitable one for acute rheumatism. At the furthest, I admit that it may be of some advantage in chronic rheumatism. When rheumatism is in the acute stage, and the pain is very violent, the slightest movement causes pain. Now the diaphoresis produced by the action of sudorifics, determines a certain degree of uneasiness, an excitement, which rather aggravate than diminish the morbid phenomena. Besides, when patients have a certain degree of perspiration, it is necessary to change their linen, which exposes them to cold, and to the production of a sudden dangerous suppression of perspiration. All these reasons united have induced me to renounce the employment of diaphoretics in the treatment of acute rheumatism, reserving them for a more advanced period of the disease, when it assumes the chronic character.

It has been customary to laud the use of tartar emetic in large doses in this affection. I have not much faith in its efficacy, in spite of the authority of Laennec, who was very partial to it. Rheumatism is very often cured by very different methods; consequently it is not necessary to seek unusual and often dangerous means. Besides, the results attributed to tartar emetic, I do not consider as sufficiently established. It is probable that Laennec, who was so partial to it, was deceived; I have not therefore, adopted this plan of treatment.

Lately a highly respectable physician has employed heroic doses of quinine, in acute rheumatism, and others have followed the example; but, far from having the success they anticipated, they met with great reverses. I have not experimented with this remedy, because, I never believed that it could exercise any beneficial influence over the disease. You know that the sulphate of quinine has two properties; one anti-periodic, the other tonic. Now in rheumatism I never could perceive any indication which demanded either of these qualities. Besides, I adhere to the rule never to experiment upon man without grave reasons, and without taking every precaution to prevent accidents. The results of the experiments made with the sulphate of quinine, have more than ever confirmed me in this circumspection. I do not consider it, moreover, as a specific remedy, and the acknowledged rules of therapeutics have been disregarded in its employment. To fully appreciate the action of a remedy but little known, you must experiment slowly and for a long time. Finally, it is neither wise nor prudent to abandon old remedies, and adopt new ones whose mode of action is unknown and which often are inferior to the old ones. Such conduct in therapeutics, besides its bad example, leads often to fearful consequences for the patients.

*Paris, June*, 1843.

